# Receptor-Mediated Enhancement of Beta Adrenergic Drug Activity by Ascorbate *In Vitro* and *In Vivo*

**DOI:** 10.1371/journal.pone.0015130

**Published:** 2010-12-13

**Authors:** Patrick F. Dillon, Robert Root-Bernstein, N. Edward Robinson, William M. Abraham, Catherine Berney

**Affiliations:** 1 Department of Physiology, Michigan State University, East Lansing, Michigan, United States of America; 2 Department of Large Animal Clinical Science, Michigan State University, East Lansing, Michigan, United States of America; 3 Department of Research, Mount Sinai Medical Center, Miami Beach, Florida, United States of America; University of Giessen Lung Center, Germany

## Abstract

**Rationale:**

Previous in vitro research demonstrated that ascorbate enhances potency and duration of activity of agonists binding to alpha 1 adrenergic and histamine receptors.

**Objectives:**

Extending this work to beta 2 adrenergic systems in vitro and in vivo.

**Methods:**

Ultraviolet spectroscopy was used to study ascorbate binding to adrenergic receptor preparations and peptides. Force transduction studies on acetylcholine-contracted trachealis preparations from pigs and guinea pigs measured the effect of ascorbate on relaxation due to submaximal doses of beta adrenergic agonists. The effect of inhaled albuterol with and without ascorbate was tested on horses with heaves and sheep with carbachol-induced bronchoconstriction.

**Measurements:**

Binding constants for ascorbate binding to beta adrenergic receptor were derived from concentration-dependent spectral shifts. Dose- dependence curves were obtained for the relaxation of pre-contracted trachealis preparations due to beta agonists in the presence and absence of varied ascorbate. Tachyphylaxis and fade were also measured. Dose response curves were determined for the effect of albuterol plus-and-minus ascorbate on airway resistance in horses and sheep.

**Main Results:**

Ascorbate binds to the beta 2 adrenergic receptor at physiological concentrations. The receptor recycles dehydroascorbate. Physiological and supra-physiological concentrations of ascorbate enhance submaximal epinephrine and isoproterenol relaxation of trachealis, producing a 3–10-fold increase in sensitivity, preventing tachyphylaxis, and reversing fade. *In vivo*, ascorbate improves albuterol's effect on heaves and produces a 10-fold enhancement of albuterol activity in “asthmatic” sheep.

**Conclusions:**

Ascorbate enhances beta-adrenergic activity via a novel receptor-mediated mechanism; increases potency and duration of beta adrenergic agonists effective in asthma and COPD; prevents tachyphylaxis; and reverses fade. These novel effects are probably caused by a novel mechanism involving phosphorylation of aminergic receptors and have clinical and drug-development applications.

## Introduction

Endogenous allosteric modulators (hereafter “enhancers”) of G-protein coupled receptors that act via extracellular sites are extremely rare [1] and none have previously been identified for aminergic receptors such as beta 2 adrenergic receptors (B2AR). B2AR control the relaxations of bronchioles in the lung. During asthma attacks, treatment involves exposing these receptors to epinephrine and related compounds such as albuterol or isoproterenol to produce dilatation and ease breathing. Persistent exposure of adrenergic receptors to agonists results in desensitization of the receptors. The short term fall-off in drug potency is called fade and probably results from the phosphorylation of the receptor, putting it into an inactive state. Longer term, desensitized receptors are internalized into lung cells resulting in a refractory response to repeated doses of adrenergic agonists, a phenomenon called tachyphylaxis. Both fade and tachyphylaxis are problems for patients with asthma and COPD. Fade limits the length of time an adrenergic agonist stays active; tachyphylaxis results in less and less effect from any given dose of drug that is repeated over time. The discovery of compounds that can enhance adrenergic agonists for asthma treatment could therefore have many benefits for peoples suffering from asthma and COPD.

The possible utility of adrenergic enhancers for treatment of asthma and COPD are several. To begin with, there is a gap in current treatment strategies for these diseases due to the types of drugs available to patients. Short acting (rescue) inhalers use drugs such as epinephrine and albuterol that act instantaneously but persist for only a couple of hours. Long acting inhalers use drugs such as formoterol and salmeterol that do not begin to have an effect for an hour or two, but persist for up to twelve hours. Short-acting and long-acting drugs cannot be used together without risk of death, so that patients must choose one approach to control of their disease or the other. Enhancers might be able to convert short acting rescue inhalers into long acting formulations that still have immediate efficacy. In addition, enhancers might be able to decrease adrenergic agonist doses significantly, thereby decreasing the side effects of beta agonists, which include increases heart rate, blood pressure, breathing rate, nervousness, and risk of heart attack and stroke. Less drug translates into less risk.

We are investigating such adrenergic enhancers. We reported previously that ascorbate can enhance the *in vitro* alpha adrenergic activity [2], [3] and histaminergic activity [4] of smooth muscle, independent of the anti-oxidant activity of ascorbate on these amines. We also demonstrated that this phenomenon is very likely receptor mediated, since ascorbate binds to the histamine receptor with a Kd equal to its ability to enhance histamine activity [4]. We report here that ascorbate, although it has absolutely no effect on smooth muscle by itself, significantly enhances beta-adrenergic-mediated smooth muscle relaxation and most likely does so by means of allosteric modulation of an extracellular site on the receptor. Using the human beta 2 adrenergic receptor, ascorbate was found to bind to an outside loop of the receptor, near the agonist binding site. The enhancement action of ascorbate is not related to its anti-oxidant activity as the effect is found with drugs that do not oxidize measurably during the course of the experiments. Both receptor sensitivity and the duration of action of beta adrenergic agonists are increased, tachyphylaxis due to repeated doses of agonists is prevented, and fade can be reversed by administration of ascorbate. Further, in addition to ascorbate assisting adrenergic receptor activity, the adrenergic receptor assists ascorbate's anti-oxidant activity. The receptor converts oxidized ascorbate, which is not useful in preventing the oxidation of other molecules, into its useful, reduced form. Thus, adrenergic receptors may also have enzymatic activity, controlling their extracellular environment. [4]. Beta 2 adrenergic enhancement was demonstrated in both healthy sheep and diseased horses, as well as in tissues isolated from pigs and guinea pigs. The ascorbate enhancement of beta 2 adrenergic potency, prevention of tachyphylaxis, reversal of fade, and the reduction of ascorbate by adrenergic receptors are all novel findings, reported here for the first time. We propose that adding ascorbate (or other aminergic enhancers that we have identified, such as ethylenediaminetetraacetic acid and opiates [2]–[4] at physiological or supraphysiological concentrations to pulmonary medications may prove highly beneficial in treating diseases such as COPD and asthma by permitting the use of lower doses of beta adrenergic agonists, thereby decreasing the unwanted side effects of these drugs. Since the ascorbate is delivered directly to the lungs, its enhancement effects on aminergic compounds are largely limited to the lungs. The novel receptor-mediated mechanism that we report may also permit the development of novel combinations treatments or new tethered drugs that will be more efficacious. Finally, the discovery of extracellular allosteric modifiers of adrenergic receptors may permit novel insights into the structures and functions of these receptor mechanisms.

## Results

**Figure 1:**
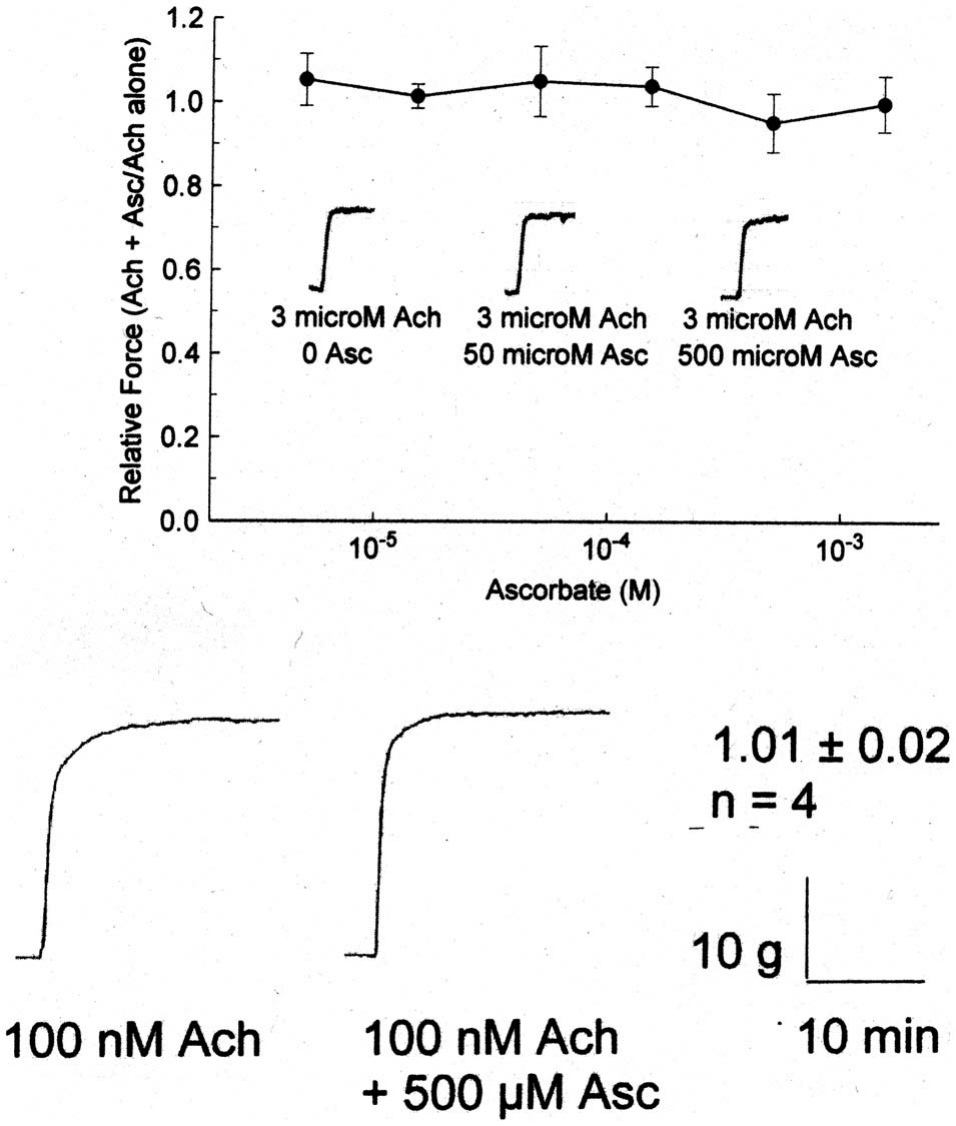
Top: Effect of ascorbate (0 to 1.5 mM) on 5 minute force of acetylcholine (3 µM) induced contractions of guinea pig trachealis. Values are mean ± SE and N = 5. No significant differences (p<0.05) from baseline (0 Asc) were observed over the 5 minute course of the contractions (exemplars imbedded in figure). Bottom: Effect of ascorbate (500 uM) on 20 min acetylcholine-induced contractions (3 uM) of porcine trachealis. No effect was observed for an N of 4. For further Asc controls, see Figure 7 (lack of effect of Asc on carbachol-induced bronchoconstriction in sheep) and Figure 9 (lack of effect of Asc on bronchoconstriction due to heaves in horses).

The effect of ascorbate on beta 2 relaxation of smooth muscle was measured. Asc at concentrations up to 500 uM by itself had no effect on guinea pig or porcine trachealis preparations contracted with acetylcholine (Figure 1). Additional control experiments in live horses with heaves and sheep treated with carbachol (see below) showed similarly that there was no measurable effect of ascorbate by itself in any preparation at any dose utilized in these experiments.

**Figure 2:**
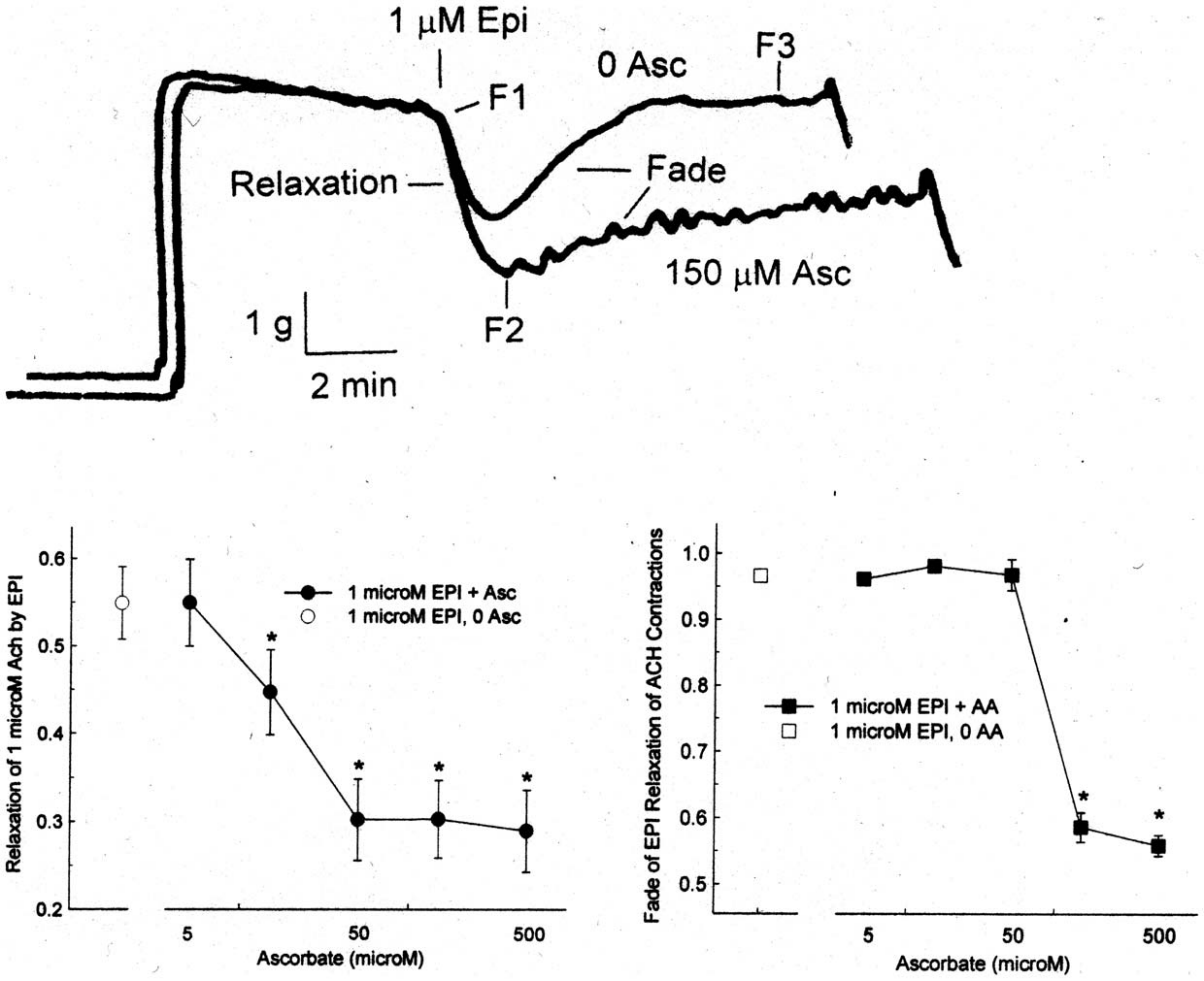
Effect of ascorbate on 1 µM epinephrine relaxation of 1 µM acetylcholine contractions of porcine trachealis. Upper panel: chart records of individual contractions with 0 and 150 µM Asc. F1 is the 5 minute Ach force, at which time Epi is added. F2 is the initial relaxation produced by Epi. F3 is the steady state force *after* the fade of the Epi relaxation. Middle Panel: Effect of ascorbate on initial epinephrine relaxation. Values are mean ± SE. The open symbol is 0 Asc; the filled symbols are 5–500 µM Asc. Note the log scale for the x axis. The * indicates a significant difference (p<0.05) from the 0 Asc relaxation. Lower Panel: Effect of ascorbate on the fade of the epinephrine-induced relaxation. Values are mean ± SE. The open symbol is 0 Asc; the filled symbols are 5–500 µM Asc. Note the log scale for the x axis The * indicates a significant difference (p<0.05) from the 0 Asc fade.

**Figure 3:**
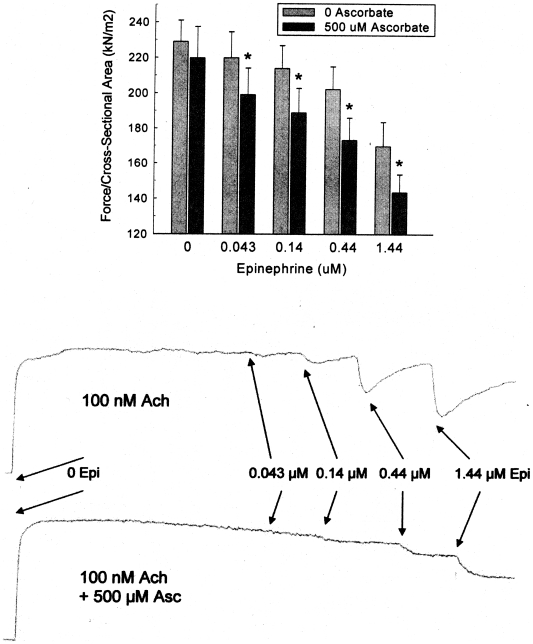
Top: Effect of 500 µM ascorbate on cumulative epinephrine relaxation (1 µM Epi) of 1 µM Ach contractions of porcine trachealis at ten minutes following each Epi addition. Values are mean ± SE. N = 4. The * indicates a significant difference (p<0.05) from the 0 Asc data. Bottom: Typical chart recordings showing the effect of of 500 µM ascorbate on cumulative epinephrine relaxation (1 µM Epi) of 1 µM Ach contractions of porcine trachealis. Asc not only increases the duration of relaxation, but also blunts the acute effects of each cumulative dose (perhaps because Asc binds to Epi [3], [23], [24], modulating its availability to the receptor).

The effect of ascorbate on beta 2 relaxation of smooth muscle was next tested in the presence of Epi. Superimposed chart records of two contractions ± 150 µM Asc are shown in upper part of Figure 2. Epi produces a significant relaxation in both cases, but the presence of Epi + Asc results in a deeper relaxation than Epi alone. Also, the recovery of force within a single contraction, defined as the fade of the relaxation effect of Epi, occurs more slowly and to a lesser degree in the presence of Asc than in its absence. The lower panel of Figure 2 shows the Asc concentration dependence of the initial relaxation (F2 in the figure) and total fade (F3 in the figure) in individual contractions, with both showing significant differences in the physiological Asc range of 40–100 µM [5]. In Figure 3, using cumulative Epi dose-response curves on Ach contractions of the pig trachealis, 500 µM Asc plus Epi produced a significantly greater relaxation than Epi alone at concentrations of Epi from 0.043 to 1.44 µM.

**Figure 4:**
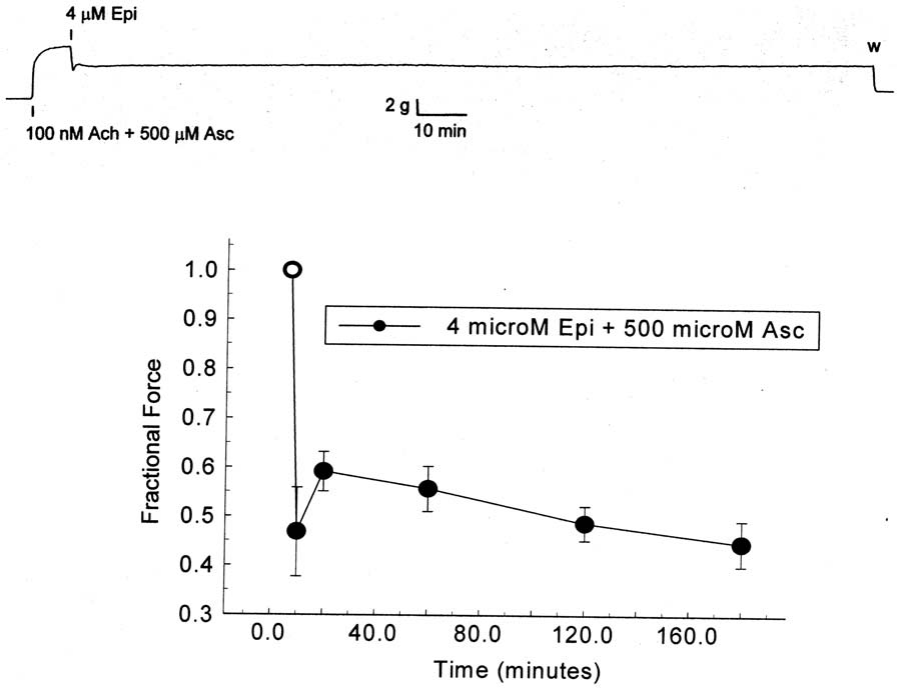
Effect of 500 µM ascorbate on prolonged 4 µM epinephrine relaxation of 100 nM Ach contraction of porcine trachealis. Upper panel: chart record of experiment. Lower panel: Values are mean ± SE. The open symbol is the relative force immediately prior to the introduction of Epi and Asc. The closed symbols represent values at different times after the introduction of Epi and Asc.

Figure 4 shows the effect of the supranormal concentration of 500 µM Asc on prolonged Epi-induced relaxation of pig trachealis Ach-induced contractions. The upper panel shows one of the chart records of this experiment. The lower panel shows the relative force at selected times thereafter. The open symbol represents the force immediately prior to the introduction of Epi. After 3 hours, during which some oxidation of Epi and ascorbate has presumably occurred (both are at the top of their dose-response ranges), the relaxation remains, with no fade occurring. Note in the chart record that as soon as the Ach, Epi and Asc are washed away, the tissue immediately relaxes to its pre-contraction level.

**Figure 5:**
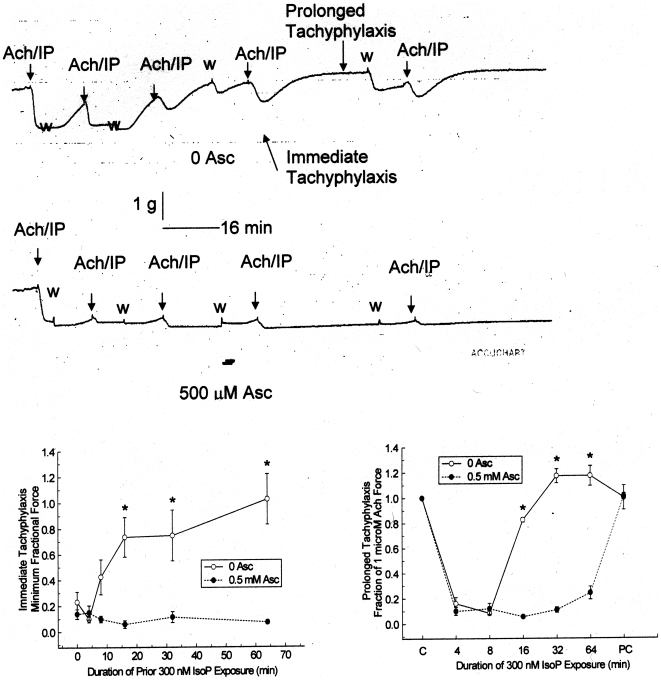
Effect of ascorbate on tachyphylaxis of 30 nM isoproterenol (IP) relaxation of 1 µM acetylcholine (Ach) contractions of guinea pig tracheal rings. Upper panels: chart records of progressive contractions in the presence of 0 and 500 µM Asc. The Ach/IP indicates the addition of Ach and IP. The w indicates the washout of Ach and IP. Lower panels: Quantitation of immediate and prolonged tachyphylaxis. The immediate tachyphylaxis measurements are plotted as a function of the duration of IP exposure of the immediate prior application. The prolonged tachyphylaxis measurements are plotted as a function of the duration of the IP application. The C (control) and PC (post-control) in the prolonged graph (lower panel) are the forces generated by the tissue prior to and following the Ach/IP applications, respectively. Values are mean ± SE. The open symbols are 0 Asc; the filled symbols are 500 µM Asc. The * indicates a significant difference (p<0.05) from the 0 Asc data.

In contrast to fade within a single contraction, tachyphylaxis is the decrease in pharmacological effectiveness in sequential exposures. In this case, the effect of Asc on the tachyphylaxis of isoproterenol (IP) was tested using guinea pig trachea. In the upper panel of Figure 5, a chart record of progressively longer contractions of Ach + IP (with 0 Asc) for 4, 8, 16, 32, and 64 minutes is shown. There are 10 minute washes between each contraction. The guinea pig trachea has spontaneous contractions during washout periods. During the first, 4 minute application of IP, there is nearly complete relaxation of the tissue. The following 8 minute application also shows nearly complete relaxation to IP. However, during the 16, 32 and 64 minute applications, the tissue does not completely relax. It exhibits both immediate tachyphylaxis, not reaching the low initial force produced at 4 and 8 minute applications, and prolonged tachyphylaxis, reaching a plateau at an even higher force. During identical experiments with 500 µM Asc included with the IP, shown in the second chart record panel, the contrast could not be greater. There is no immediate or prolonged tachyphylaxis to IP. The quantitation of this effect is shown in the lower panels of Figure 5. Both the lowest force following IP application, the measurement of the immediate tachyphylaxis, and the highest force during the IP application, the measurement of prolonged tachyphylaxis, are significantly different between the 0 and 500 µM Asc applications. Importantly, during post control (PC) contractions after the removal of both IP and Asc, both conditions return to the control (C) force, indicating that neither application damaged the tissues.

**Figure 6:**
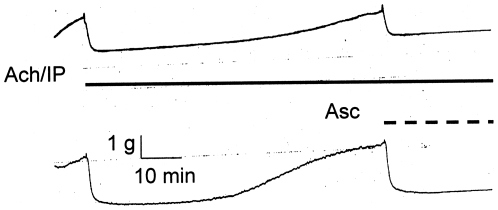
Ascorbate (500 µM) reversal of the fade of 30 nM isoproterenol relaxations of 1 µM Ach contractions of twp separate guinea pig tracheal rings. The solid horizontal line indicates duration of Ach/IP application. The dashed line indicates the application of Asc.

Figure 6 shows that in addition to blocking IP tachyphylaxis, Asc also reverses the fade of IP-induced relaxation of the guinea pig trachea. Both chart records show the same experiment, in which Ach and IP application produces a profound relaxation of the trachea, but after 73 minutes, the relaxation has faded. The addition of Asc, while Ach and IP are still present, renews the relaxation of the trachea. Quantitatively, for N = 7, the 73 minute fractional force is not significantly different (0.81±0.15, SE, p = 0.24) from the force (1.0±0.0) immediately before IP was first applied. Both the lowest IP force (0.05±0.03) and the force following Asc (0.04±0.02) are significantly different from the initial application force (p = 2×10^−8^ and 7×10^−8^, respectively), but are not different from each other (p = 0.86).

**Figure 7:**
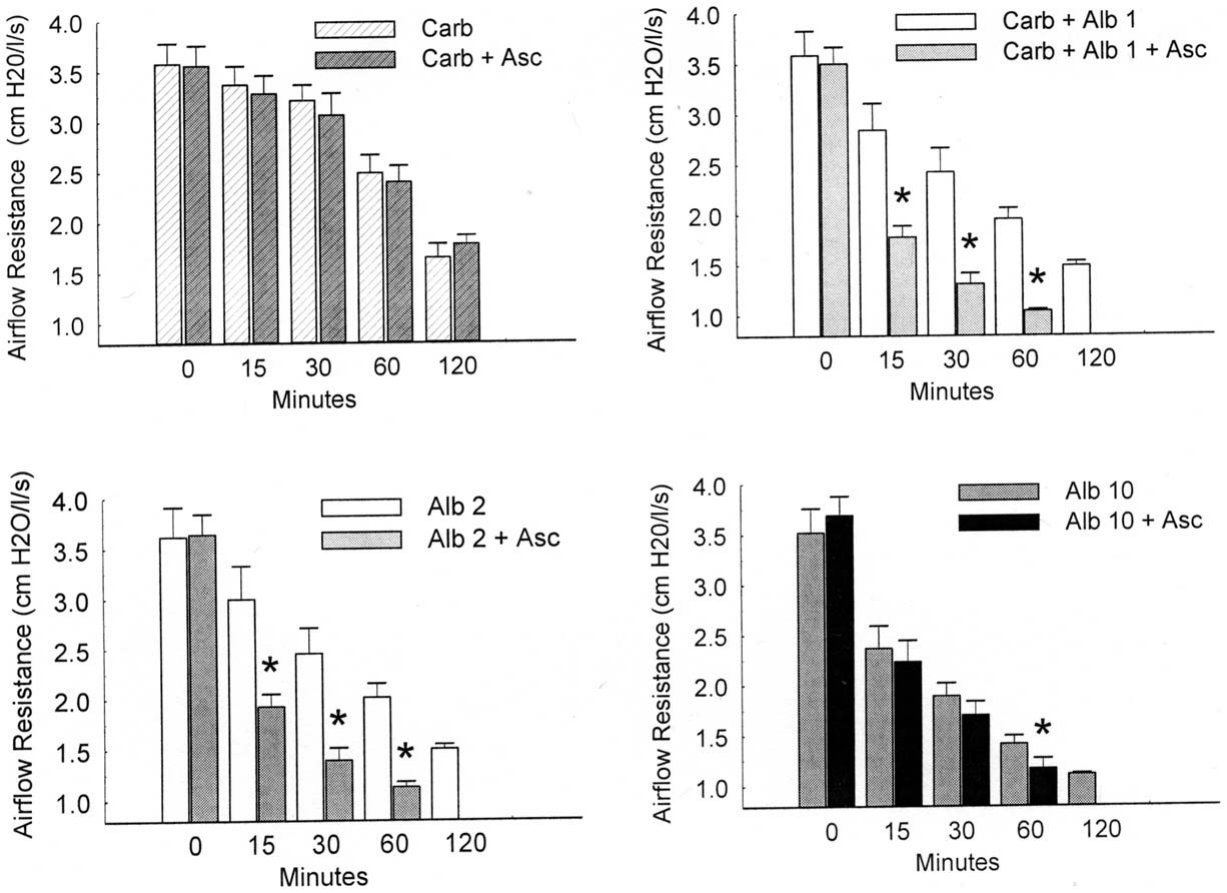
Effect of ascorbate on the airflow resistance of intubated sheep. Sheep bronchioles were contracted by carbachol and relaxed with albuterol. Values are mean ± SE. The graphs from upper left to lower right are for 0, 1, 2 and 10 breaths of albuterol in the presence and absence of Asc. The * indicates a significant difference (p<0.05) from the 0 Asc data.

Following the *in vitro* experiments, we began *in vivo* measurements of the effect of ascorbate on beta 2 relaxation of pulmonary smooth muscle with a proof of concept study in horses with the obstructive airway disease heaves. Baseline Δ Ppl_max_, a simple indicator of lung function than can be made in untrained horses, did not differ before vehicle or with Asc treatment alone (mean ± SE, 49.4±7.9 cm H_2_O). After inhalation of vehicle, albuterol decreased the Ppl_ratio_ in a dose-dependent fashion with maximal effect achieved at a dose of 360 micrograms (Figure 7). Pretreatment with ascorbate significantly potentiated the response to albuterol. In cumulative dose response controls, Asc (0, 5, 10, and 15 mg/ml) showed no significant effect on Ppl_max_. Administration of atropine (0.02 mg/kg IV) after ascorbate significantly decreased ΔPpl_max_ to 20.3±4.3 cm H_2_O within 15 minutes indicating that the horses had the reversible airway obstruction typical of heaves (data not shown).

**Figure 8:**
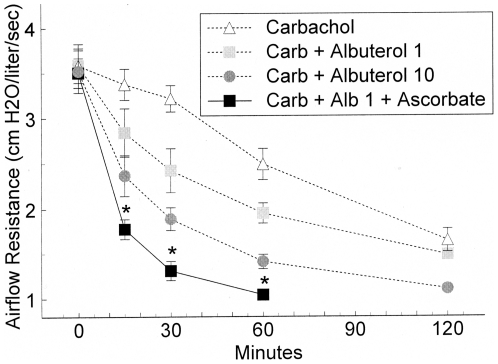
Comparison of effect of 1 breath of albuterol with ascorbate with 0, 1 and 10 breaths of albuterol on carbachol induced increases in airway resistance. Values are mean ± SE. The * indicates a significant difference (p<0.05) from the 0 albuterol control. The + indicates a significant difference of the 1 breath of albuterol with ascorbate from the 10 breaths of albuterol.

**Figure 9:**
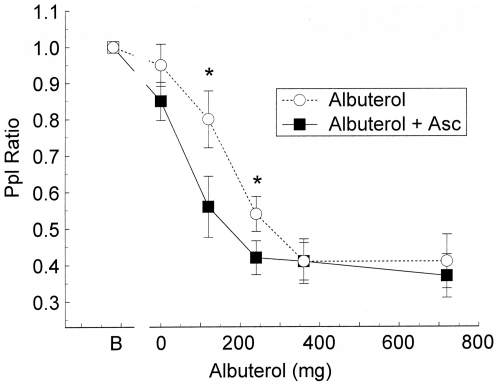
Effect of ascorbate on albuterol relaxation of breathing resistance in horses with heaves. Values are mean ± SE. The B indicates the baseline measurement of breathing resistance. Open circles are measurements of resistance with 0 Asc; filled squares are measurements with ascorbate. The * indicates a significant difference (p<0.05) from the 0 Asc data.

Based on these encouraging results from horses with heaves, the in vivo effect of Asc was investigated more extensively in sheep during carbachol-induced airway constriction and albuterol relaxation (Figure 8). In the control trial of Asc without albuterol in the upper left panel, the airflow resistance was not significantly different from the carbachol alone challenge at any time point. In the upper right panel the presence of Asc in 1 breath of albuterol produced a significantly lower airflow resistance when compared with albuterol alone. Similar results with 2 breaths of albuterol are shown in the lower left panel, with Asc improving the ease of breathing. No measurement of albuterol + Asc was made *at 120 minutes* for either 1, 2 or 10 breaths. The lower right panel shows that for the nearly maximal relief produced by 10 breaths of albuterol, Asc did not produce a significant difference in relief as measured at times less than 60 minutes. Figure 9 shows the grouped data for carbachol alone, carbachol +1 breath of albuterol, carbachol +10 breaths of albuterol, and carbachol +1 breath of albuterol + Asc. Ascorbate, which had no effect on carbachol treatment by itself, has a significantly lower airflow resistance with 1 breath of albuterol + Asc than the resistance of 10 breaths of albuterol alone. The addition of Asc with the lower level of albuterol produces more relief than having 10-fold more albuterol.

**Figure 10:**
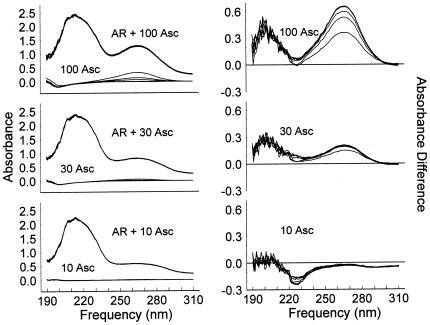
Spectroscopic measurements of ascorbate-human adrenergic receptor binding. The figures on the left show the superimposed records of six spectra collected 118, 141, 155, 188, 199, and 228 minutes after the samples were prepared. Samples with both AR and ascorbate are indistinguishable from one another and do not change over time, while the ascorbate alone samples show ascorbate oxidation and a decrease in absorbance in the 266 nm range. The difference spectra in the right hand column show an ascorbate concentration dependent increase between 190 and 210 nm, an absorbance decrease between 220 and 230 nm at 10 µM ascorbate, and the difference between the oxidizing ascorbate solutions without AR and the non-oxidizing ascorbate with AR in the 260–270 nm range.

Having established the in vitro and in vivo ascorbate enhancement of beta 2 adrenergic relaxation of smooth muscle, the mechanism of action of ascorbate on the beta 2 receptor was investigated. Previous research had demonstrated that Asc binds directly to the histamine receptor on the first extracellular loop [4], so our hypothesis was that a similar extracellular loop on the B2AR might also exist. Figure 10 shows the accumulated spectra of the solutions with the different Asc concentrations in the presence and absence of human beta 2 adrenergic receptor on the left, and the difference spectra (Asc and AR combined minus individual Asc and AR solutions) on the right. In the unprocessed spectra on the left, six successive spectra over a period of more than one hour are superimposed as the AR +100 µM Asc, AR +30 µM Asc, and AR +10 µM Asc lines. The spectra are so consistent that the individual AR + Asc spectra cannot be distinguished. Note that in the 100 Asc (with no AR), and to a lesser extent in the 30 Asc (with no AR), series of lines, the superimposed spectra are distinguishable, with the Asc absorbance centered at 266 nm disappearing with time. The difference spectra on the right show three distinct regions. At 266 nm, the six difference spectra containing the AR + Asc are again superimposed, forming the thicker upper line in each series. The lower lines, most clearly seen in the 100 Asc series, are produced as the Asc is oxidized in the absence of AR. There is a decrease in the absorbance at 220–230 nm in the 10 µM Asc series, indicating Asc binding to the AR at this sub-physiological concentration. In the region centered at 205 nm, there is an Asc concentration dependent increase in the absorbance, indicating a second binding of Asc, with this binding in the physiological Asc concentration range. Note that at 30 and 100 µM Asc, the presence of 1.26 µM AR is able to virtually stop the oxidation of Asc.

The data obtained from the AR-Asc experiments just described were compared with identical experiments involving untransfected cell membranes and AII-transfected cells membranes, previously published in Dillon, et al. [4]. Asc binding to AR-transfected membranes was more than five times that observed with the untransfected and AII-transfected membranes. The decreased binding was associated with significantly increased Asc oxidation rates in the untransfected cell preparations compared with the transfected ones [4].

**Figure 11:**
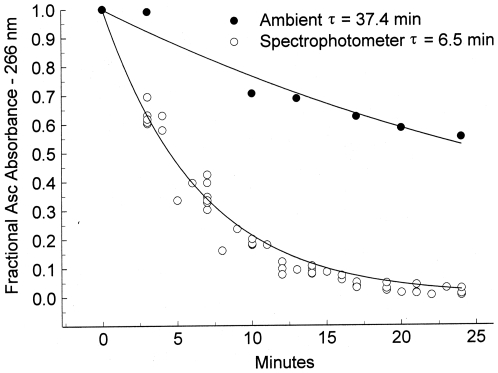
Quantitation of ambient (room) and spectrophotometer (UV light exposed) oxidation of ascorbate. Each symbol represents the average absorbance at 266 nm of 3 wells to which ascorbate is added. The additions were temporally sequential, with both the new additions and those from previous additions measured separately at each subsequent time. The solid symbols, determined by the first measurement after the addition from a room stock, show the ambient Asc oxidation. The open symbols show the Asc oxidation of the seven sets of three wells within the spectrophotometer, with the data normalized to the initial absorbance of that sample when first measured in the spectrophotometer. The lines were calculated from a linearized least squares fit of the data with an absorbance of 0.3 or greater of the initial absorbance.

Using the initial addition of seven sets of three wells, the ambient ascorbate oxidation rate constant was 37.4 minutes, with a correlation coefficient of 0.96 (Figure 11). For samples exposed to the UV light of the spectrophotometer, average rate constant is 6.5 minutes, with a correlation coefficient of 0.97. The exposure of ascorbate to the UV light inside the spectrophotometer increases the oxidation rate 6-fold. The presence of transfected B2AR prevents the oxidation of Asc under both conditions.

**Figure 12:**
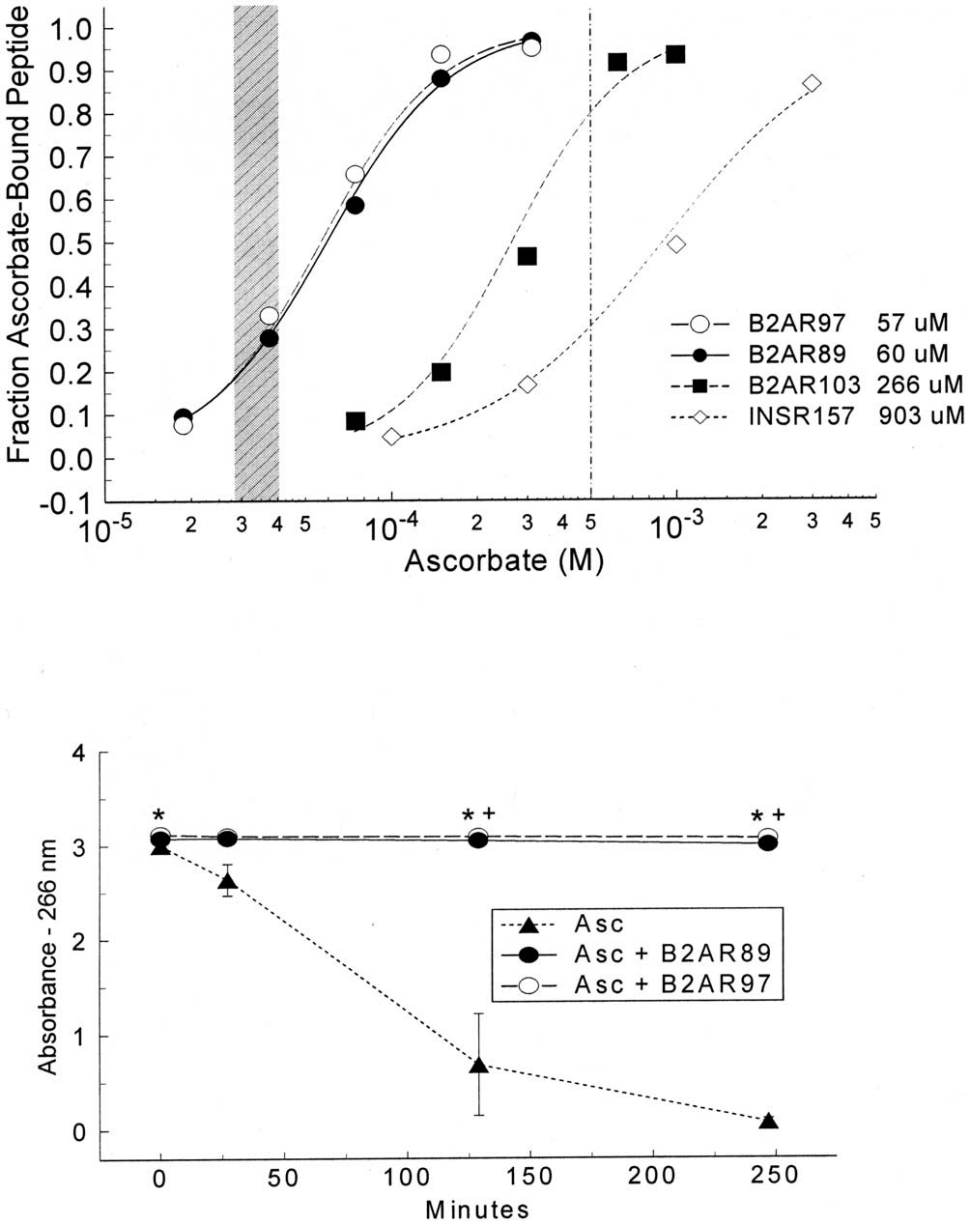
Beta 2 Adrenergic Peptides and Ascorbate. Upper panel: binding ascorbate to beta 2 adrenergic receptor and insulin receptor peptides. The peptide sequences are listed in Table 1. The initial amino acid number of the peptide is listed in the legend. The shaded area represents the range of the respiratory system concentration of ascorbate in humans (28–40 µM). The vertical line represents 500 µM ascorbate, the highest Asc concentration used during *in vitro* experiments herein. Lower panel: prevention of ascorbate oxidation by AR peptides. The * indicates a significant difference (p<0.05) between the Asc alone absorbance and the Asc absorbance in the presence of the peptide.

The binding of ascorbate to peptides with sequences from the first extracellular loop of the beta 2 adrenergic receptor also prevents or retards oxidation, as is shown in Figure 12 and Table 1. All three peptides spanning the loop (amino acids 89–99, 97–106, and 103–113) showed significant Asc affinity, two centered within the normal lung Asc range and the third with greater sensitivity than the highest Asc concentration used in the isolated tissue experiments. Three peptides with sequences from the insulin receptor were used as controls. One (157–166) had modest ascorbate binding, but still weaker than those from the adrenergic receptor. Two additional insulin receptor peptides (392–404, 425–433) had much lower Asc affinity, with estimated K_d_s of >1500 and 3000 µM. The 89–99 and 97–106 peptides prevented the oxidation of Asc. Control peptides did not significantly retard or prevent Asc oxidation.

**Table 1:** UV spectroscopy was used to determine the binding constants of ascorbic acid binding to various synthetic peptides derived from the first extracellular loop of the human beta 2 adrenergic receptor (B2AR).

Peptide	Sequence	K_da_	R^2^	K_d_
		(µm)*		(µm)
D1DR 89–100	FWPFGSFCNIWV	>3000	estimated	
B2AR 89–99	FGAAHILMKMW	60	0.998	22
B2AR 97–106	KMWTFG**NFWC**	57	0.96	19
B2AR 103–113	**NFWC**EFTSID	266	0.968	232
INSR 157–166	TIDWSRILDS	903	0.995	861
INSR 392–404	SGYLKIRRSYALV	>1500	estimated	
INSR 425–433	YSFALDNQN	>3000	estimated	

**Figure 13:**
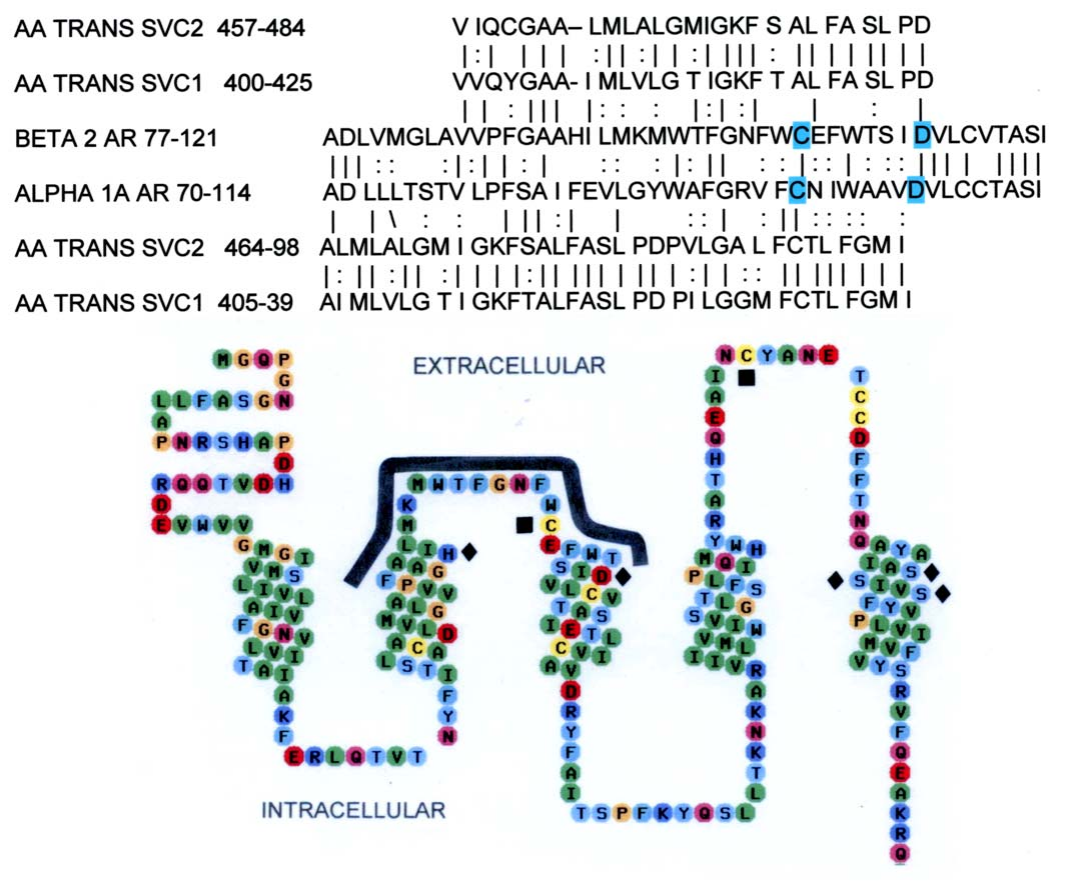
Adrenergic receptor amino acid sequences. Upper panel: adrenergic receptor and sodium-dependent ascorbate transporter homology. The amino acids of the human beta 2 AR and human alpha 1A AR are compared with the human SVC1 and SVC2 ascorbate transporters. The solid lines indicate exact matches, and the: indicates similar amino acid substitutions. Lower panel: 2-D model of relative amino acid positions in the N-terminal section of the human beta 2 AR. The solid squares indicate the cysteines that must form a disulfide bond in order for epinephrine to bind, and the solid diamonds indicate the amino acids that form the epinephrine binding site. The thick solid line indicating the ascorbate-binding site is in close proximity to the both the cysteines and the epinephrine binding site. Peptides listed in Table 1 were derived from the homologies displayed here.

This loop had been identified as being homologous to the sodium-dependent ascorbic acid transporter (Figure 13), and therefore likely to bind ascorbate. Control peptides were derived from the human insulin receptor (IR) and human dopamine D1 receptor, which lacks homology at this site for the ascorbic acid transporter. The K_d_ is the binding constant. The K_da_ is the ascorbate concentration where ½ of the peptide is bound. Rate constants were calculated from a linearized least-squares fit to an exponential decay where R^2^ is a measure of the fit of the calculated equation to the data. The highest affinity peptides share only one short sequences, which KMW, suggesting that this motif is the main component of the Asc binding site. Peptides that lack this motif have little or no Asc binding.

*K_da_ is the ascorbate concentration where ½ of the peptide is bound.

## Discussion

The data above support four novel conclusions. Although ascorbate has absolutely no effect on airway smooth muscle at any concentration tested or in any in vitro or in vivo experiment performed, (1) ascorbate increases the potency of multiple beta 2 adrenergic agonists across a range of species; (2) ascorbate prevents tachyphylaxis induced by multiple doses of beta 2 adrenergic agonists; (3) ascorbate can reverse fade associated with long term exposure to beta 2 adrenergic agonists; and (4) the beta 2 adrenergic receptor and peptides derived from its first extracellular protect ascorbate against oxidation, prolonging its effects. These observations suggest that the B2AR activity can be enhanced allosterically by Asc through a novel mechanism that may provide means to improve the treatment of asthma and COPD.

The experiments we report here must be interpreted as a *set*, each building on previous results. The most important observation is that ascorbate has no measurable effect on smooth muscle contractility, on relaxation, or on lung function. The smooth muscle experiments (Figure 1), those on sheep airway function (Figure 7), as well as in the data concerning horses with heaves described in the text, demonstrate that ascorbate has absolutely no measurable effect on uncontracted, ACh-contracted, or carbachol-contracted airway smooth muscle *in vitro* or on lung function *in vivo* in four different animal species. We previously demonstrated that ascorbate has no measurable effect at concentrations up to 500 µM on uncontracted rabbit aorta [2]–[4].

The absence of ascorbate effect by itself sets the stage for demonstrating differences in the effects of adrenergic agonists when they are in the presence of ascorbate compared to when they are not. We have demonstrated in great detail in previous papers that the enhancement effects of ascorbate cannot be due to its antioxidant properties [2]–[4]. Given the stability of the isoproteronol and albuterol used in many experiments reported here, oxidation cannot be a factor within the time courses of these experiments and ascorbate cannot be producing its enhancing effects through prevention of drug oxidation. Ascorbate also has no effect on acetylcholine- or carbachol-induced contractions, nor, as we have shown in previous papers on potassium-induced or angiotensin II-induced contractions [2]–[4]. Logically, the possible mechanisms by which ascorbate's enhancement effects are produced on smooth muscle must be limited to effects on the adrenergic system that do not share contractile or relaxile pathways shared with these other agents. These possibilities are further limited by the fact that we [2], [3] and Herlihy, et al. [6] have demonstrated previously that ethylenediaminetetraacetic acid (EDTA) also acts as an enhancer on the adrenergic system. EDTA is so polar that it cannot enter cells and has no known receptor or transporter. Since EDTA and ascorbate produce the same effects, but only one of them can enter cells, we conclude that the enhancement mechanism is an extracellular one. The only plausible extracellular mechanism that would permit both ascorbate and EDTA to produce the same enhancing effect only on the adrenergic system and only when it is activated, is through the adrenergic receptor itself.

The effects of ascorbate enhancement are surprising. The experiments using pig trachealis show that ascorbate lessens, and at sufficiently high concentration, prevents fade of epinephrine-induced relaxation of Ach contractions. The ability of ascorbate to enhance the relaxation occurs within a 100-fold epinephrine concentration range. Ascorbate also reverses the fade of isoproterenol-induced relaxation of Ach contractions of guinea pig tracheal rings, implying that ascorbate will deepen and prolong the beta agonist effects. Further, guinea pig experiments on sequential contractions show that ascorbate effectively blocks beta 2 adrenergic tachyphylaxis. Both fade and tachyphylaxis reflect down regulation of receptor, suggesting that ascorbate prevents down regulation of beta 2 adrenergic receptors and may, under some conditions, reverse it (Figures 4, 5, 6).

We suspect that such prevention and reversal of down regulation has been observed by those studying down regulation, but overlooked due to the methods used in most studies. One set of experimenters study down regulation by exposing cells or receptors to beta adrenergic agonists in the absence of ascorbate or EDTA [7], or utilize EDTA in receptor isolation but eliminate it during the preparation of the receptor for assay [8], [9]. These studies utilize concentrations of adrenergic agonists at the top of their dose response curves or near the highest end of the ranges that these drugs would be used therapeutically (10 µM isoproterenol [8]; 400–700 nM for (-)-epinephrine, 150–300 nM for (-)-isoproterenol, [9]; 1 uM isoproterenol [7]). Such concentrations are very close to the concentrations used in our experiments to produce fade and tachyphylaxis in the absence of ascorbate. In the absence of ascorbate or EDTA, such experiments will show no prevention or reversal of down regulation. The second type of down regulation studies employ ascorbate or EDTA in the assay procedure at concentrations roughly equivalent (usually 300 µM −5 mM) to those that prevent tachyphylaxis and reverse fade in our studies and yet down regulation still occurs. Crucially, these down-regulation studies employ extraordinarily high concentrations of adrenergic agonists that are 30 to 300 times greater than the concentrations employed in our experiments (e.g., up to 100 µM EPI [10], [11]; 10 µM isoproterenol [12], [13], or 100 µM isoproterenol [13]). Such huge concentrations of beta agonists overwhelm any protective effect of ascorbate or EDTA. The only group studying down regulation that does not use these very high concentrations of beta agonists in the presence of (300 uM) ascorbate employs 1 uM isoproterenol so that one might expect to observe protection against down regulation. Such protection does not occur, however, because these investigators add 1 mM of the phosphodiesterase inhibitor RO-20-1724 to their preparations [14]–[16]. Since phosphorylation of the receptor is the first step in down regulation, and phosphodiesterase prevents or reverses this down regulation, it is logical to conclude that the antiphosphodiesterase compound prevents the up-regulating effects of ascorbate. These experiments suggest that ascorbate and EDTA activity may be mediated by putting the receptor in a conformation that prevents phosphorylation.

Studies on down regulation indicate that the effects of ascorbate are not a function of its concentration per se, but of the ratio between its concentration and that of the beta agonist it is enhancing. A dose relationship between ascorbate and adrenergic agonists also exists for in vivo enhancement. In healthy sheep with chemically-induced bronchoconstriction, and in heaves-diseased horses, ascorbate inhalation improved sensitivity to the beta 2 agonist albuterol but only at submaximal doses of albuterol. The respiratory system has a lower normal ascorbate concentration range, 28–40 µM [17], than in plasma, 40–100 µM [5], presumably due to a higher oxidation rates and inflammatory processes of disease. This decrease in endogenous ascorbate may make the lungs particularly amenable to ascorbate treatment in asthma and COPD.

In light of the receptor mechanism suggested above for Asc enhancement of adrenergic function, and since Asc modifies dopamine binding to its receptors [1], [18] and binds to the first extracellular loop of the histamine receptor [4], it was logical to search for an Asc binding site on the AR that could act allosterically to modify receptor sensitivity. The main technique used for these studies was change in spectroscopic absorbance, which is routinely used to measure binding of molecules. Experiments involving the binding of Asc directly to isolated receptor preparations show that the lowest Asc concentration, 10 µM, produces an absorbance change at 220–230 nm (Figure 10). There is a slight inhibition of alpha adrenergic activity at this concentration in rabbit aortic tissue [2], not seen in the beta adrenergic experiments. The binding at physiological concentrations and higher (ca. 50 uM) occurs in the 190–210 nm range.

**Figure 14:**
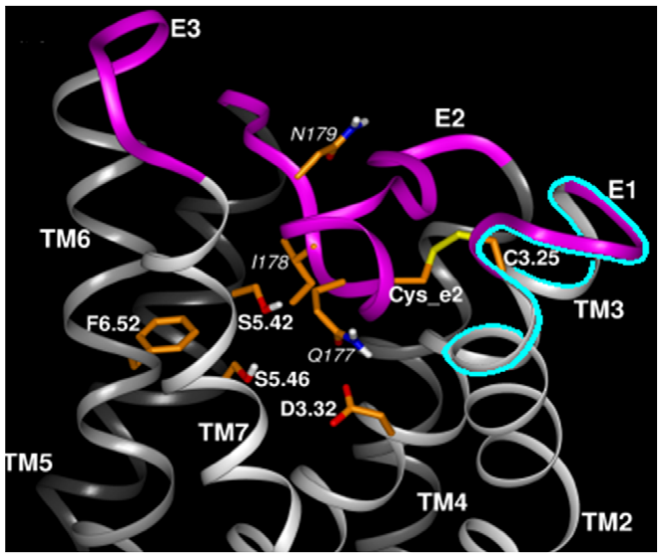
3-dimensional model of the ascorbate binding region of the adrenergic receptor. The position of the ascorbate binding site (highlighted in blue beneath the arrow) is immediately adjacent to the adrenergic binding site. (Model provided by, and adapted, with permission from Lei Shi and Jonathan A. Javitch, Columbia University College of Physicians and Surgeons, and reprinted from the Annual Review of Pharmacology and Toxicology, volume 42 © Annual Reviews www.annualreviews.org) [22].

The presence of a single homologous region shared by Asc transporters and three classes of aminergic receptors that are enhanced by Asc (Figure 12, upper panel) suggested that extracellular loop regions of the receptors might be responsible for their Asc sensitivity. The location of the binding site also provides a plausible mechanism by which Asc enhances AR activity. Epi binding to AR is mediated by a series of specific amino acid interactions involving His 79, Asp 113, Ser 203, Ser 204 and Ser 207 [19], [20]. The ascorbic acid transporter-like region is immediately adjacent to the adrenergic binding site and overlaps the disulfide bond between Cys 170 (second loop) and Cys 106 (first loop) that is required to form the high affinity form of the receptor (Figures 13, Figure 14) [20]–[22]. Ascorbic acid may help to maintain this critical disulfide bond by protecting it from oxidation, and it may further define the molecular structure of the adrenergic binding site itself, perhaps trapping agonists in the pocket formed with the second extracellular loop [22]. The stabilization of the Cys 106 residue by polarization of nearby amino acid residues helps form the high affinity state of the beta 2 AR [21]. The presence of an ascorbate binding site immediately adjacent to, and perhaps overlapping, this disulfide bond represents a logical and novel mechanism for mediating high affinity receptor activity.

Normally, upon binding an adrenergic agonist, the AR is rapidly desensitized on the order of seconds to minutes by phosphorylation carried out by beta adrenergic receptor kinase (βARK) [7]–[16]. βARK is activated by conformational changes in the AR upon agonist binding [7]–[16]. We propose that Asc binding may prevent the conformational change in the AR receptor necessary for βARK activation, thereby maintaining the AR in its high sensitivity conformation for extended periods of time. Experimentally, Asc should therefore significantly retard agonist-stimulated phosphorylation of the AR, as has previously been demonstrated for combinations of agonists with antagonists [23] and chronic beta-blockers [24]. Moreover, it is possible that once the AR is phosphorylated, binding of Asc to it (in the presence of agonist) may permit a receptor conformation modification that causes rapid dephosphorylation of the receptor, returning it to its high sensitivity state. The necessity to employ a phosphodiesterase inhibitor in order to measure desensitization by normal concentrations of adrenergic enhancers in the presence of ascorbate [14]–[16] can be interpreted as evidence that ascorbate indeed does prevent and even reverse phosphorylation of the receptor. Such a mechanism would explain our ability to reverse fade rapidly (Figure 6) since desensitization due to phosphorylation takes only a matter of seconds and can presumably be reversed on the same time scale. Notably, Parra, et al. [25] have also reported immediate reversal of fade in rabbit aortic smooth muscle preparation stimulated with adrenergic agonists and enhanced with an opioid agonist, and several groups have reported reversal of tachyphylaxis in human patients with steroids e.g. [26], [27].

The receptor spectra also reveal the striking finding that the receptor can either prevent Asc oxidation or possibly recycle dehydroascorbate back into Asc. As Figure 10 shows, ascorbate up to 100 µM does not oxidize when AR is present at 1.26 µM, even under UV spectroscopic conditions where Asc is oxidized more rapidly than under ambient conditions. The concentration difference between ascorbate and AR is so great that simple binding alone cannot explain the prevention of ascorbate oxidation. Ascorbate must be reduced by AR at a rate faster than the rate of oxidation (Figure 10). We infer that the adrenergic receptor, like the histamine receptor [4] may have enzymatic activity, in this case controlling the oxidation-reduction state of its extracellular environment. This oxidation-reduction cycle between Asc and the first extracellular loop mimics the well-characterized oxidation-reduction cycle of Asc with glutathione, another Cys-containing peptide [28]. We are currently characterizing this ascorbate-AR peptide oxidation-reduction system in much more detail and preliminary data show that peptides derived from the first extracellular loops of B2AR and the histamine receptor are able to recycle Asc just as efficiently as glutathione on a mole-to-mole basis (unpublished data). The binding of Asc to Epi [2], [4], [29], [30] and to specific amino acid residues would form an evolutionarily advantageous integrated system of interactions useful to AR receptor activation and to maintenance of reduced ascorbate. It is intriguing to consider the possibility that other such antioxidant or vitamin-associated receptor systems linking agonist-cofactor interactions may exist.

We note that the adrenergic receptor-mediated mechanism for Asc enhancement investigated here may be supplemented by other mechanisms. In addition to smooth muscle contraction, closure of the periphery of the lung has been reported to an important factor in asthma in humans [31] and mice [32]. Moreover, beta agonists may reduce lung vascular permeability and increase secretion of lung surfactants [33], [34]. The present study did not examine closure of the periphery, lung vascular permeability or lung surfactant secretion, so future studies will be needed to explore the degree to which ascorbate enhances or alters these phenomena.

We stress that no adverse effects of ascorbate inhalation were observed in either horses or sheep during or after our experiments. Moreover, there is good reason to believe that the clinical enhancement of albuterol observed in our experiments is largely or completely limited to the trachea and bronchioles. Although some ascorbate will be absorbed into the blood stream, just as some albuterol is absorbed, the effect of the additional ascorbate on endogenous concentrations of ascorbate will be inconsequential. Even if all of the ascorbate delivered via the lungs to a horse or sheep were to be absorbed into the blood stream instantaneously, the overall rise in serum ascorbate would be less than two percent, which would have negligible effects on systemic aminergic function. Thus, the sheep data (Figures 7 and 8) suggest that ASC may allow the dose of adrenergic drug to be dropped more than ten fold, retaining therapeutic efficacy, but also reducing systemic side effects such as the increased blood pressure, increased heart rate, nervousness, and sleeplessness that often accompany adrenergic inhaler use.

Both carbachol-treated sheep and horses with heaves are well-characterized models for human airway disease so that the results reported here may have practical applications to the treatment of human asthma, chronic obstructive pulmonary disease, and related conditions. In particular, an Asc-beta adrenergic agonist combination may fill a current therapeutic need. Existing beta adrenergic drugs for asthma and COPD are either quick onset, short-duration drugs (e.g., albuterol and epinephrine) used as “rescue inhalers”, or slow onset, long-duration drugs (e.g., formoterol and salmeterol) that do not address an ongoing asthma attack. Our data suggest the possibility of producing quick-onset, long-duration drugs [2]–[4], [35]. We believe that such enhanced drugs might avoid some of the serious health risks posed by formoterol and salmeterol. In our view, the problem with current long-acting beta-agonists is two-fold: 1) they have very slow onset leaving asthmatics open to acute asthma attacks during the first couple hours after treatment; 2) these acute asthma attacks cannot then be treated effectively because immediate onset adrenergic agonists (rescue inhalers) synergize with the slow-onset, long-acting ones greatly increasing risk of stroke and heart attack. Enhanced formulations [2]–[4], [35] of quick onset beta agonists may avoid these risks by maintaining their quick onset (Figures 7 and 8) yet producing long-acting effects (Figures 3 and 4). Moreover, it may be possible to use an ascorbate inhaler to supplement adrenergic inhalers, or even intravenous ascorbate in the cases of hospitalized patients resistant to adrenergic treatment, to prevent tachyphylaxis or to reverse fade (Figures 5 and 6). The observation that enhancement is accompanied by prevention of tachyphylaxis and reversal of fade suggests further that it might be possible to formulate novel therapeutics that do not have the rebound effect (rhinitis medicamentosa) that current adrenergic decongestants display, or that obviate the paradoxical pharmacology documented by Bond [24] with regard to the use of beta blockers in asthma.

In addition, the discovery of Asc enhancement of adrenergic activity may be of utility beyond airway disease since adrenergic drugs have a wide range of medical uses. The Asc binding site characterized here on the human B2AR is conserved in all AR types (Figure 12), the histamine receptor [4] and dopamine receptors, and in all mammalian species sequenced. We have already demonstrated similar enhancement of alpha-adrenergic-induced contractile effects on aortic preparations *in vitro* [2], [3] and of histamine receptor-mediated contractile effects on aortic preparations *in vitro* [4]. Data from human drug trials show that ascorbate produced a three-fold increase in the inotropic effect of dobutamine [36]; a two- to three-fold increase in intraduodenal isoproterenol activity [37]; a similar increase in vasodilation caused by endogenous catecholamines in veins [38]; and an increase in phenylephrine induced cardiovagal baroreflex sensitivity in older men [39].

One problem that may attend the *systemic* delivery of Asc or other enhancers (as opposed to direct delivery to the nasal mucosa, sinuses, or airway) is the risk of enhancing all endogenous amines, which may create risks such as high blood pressure, tachycardia, and stroke. We have demonstrated that phenylpropanolamine taken with megadoses of oral Asc could produce sufficient increases in smooth muscle contraction to be fatal [2]. Thus, tethered compounds that link Asc or other enhancers to specific aminergic drugs so that enhancement is mediated only at specific receptor subtypes will be a preferred direction for the development of future drugs [35].

In sum, we report a novel receptor-mediated enhancement of beta adrenergic drugs that increases duration of drug activity and receptor sensitivity, prevents tachyphylaxis, reverses fade, has clear clinical applications to the treatment of asthma and COPD, and has wider drug development implications for many drugs targeted at aminergic receptors. We propose that these effects are mediated by a novel mechanism.

## Materials and Methods

All experiments involving animals were approved by the appropriate university animal use committee: Michigan State University for pigs, guinea pigs and horses, the University of Miami for sheep.

### Isolated tissue experiments

Physiological salt solution (PSS) contained (in mM) 116 NaCl, 5.4 KCl, 19 NaHCO_3_, 1.1 NaH_2_PO_4_, 2.5 CaCl_2_, 1.2 MgSO_4_, and 5.6 glucose. PSS was aerated with 95% O_2_-5% CO_2_ to maintain pH 7.4 and warmed to 37°C before addition to tissue baths. Distilled and filtered water with a resistance of 17 MΩ was used for all experiments. Smooth muscle strips were prepared from porcine trachealis collected at slaughter. The trachealis was cut from the trachea, the epithelium and lamina propria were pealed away, and 10×3×2 mm strips cut with a dissecting scissors and a single edge razor blade. The strips were attached to a micrometer and force transducer using Plexiglas-stainless steel clips and mounted in tissue baths. [2] The strips were bathed in physiological salt solution (PSS) at 37°C and bubbled with 95% O_2_/5% CO_2_. After the strips were mounted, each was stretched to 5 g and allowed to stress relax for 2 h before activation. If stress relaxation reached 0 g, the ring was restretched to 2 g and allowed to stress relax until the passive force was stable. This procedure leaves the strips near their optimal length for force development. Then, prior to any experimentation, each strip was pre-contracted with K+ for at least 10 min, at which time prewarmed PSS was used to wash out the contracting solution. Relaxation to baseline typically took 10 mins or so. In addition, before experimentation began, each strip was contracted with a prewarmed 3 µM solution of ACh. This procedure was repeated at the ending of each day's set of experiments to ensure that force contraction was maintained stably throughout the experiments. Strips that did not respond appropriately to K+ or ACh initially were discarded and data from strips that did not maintain the same degree of ACh contractility across an entire set of experiments were not used.

Acetylcholine (Ach) and ascorbate (Asc) were prepared as refrigerated stocks in PSS until mixed for pre-warming and aeration immediately prior to introduction into the tissue baths. The strips were contracted, as shown in Figure 1, with 1 µM or 100 nM Ach ± different concentrations of Asc. After 5 minutes, 1 µM Epi was added by syringe from a 1 mM refrigerated stock solution. For the guinea pig experiments, 300–400 g male animals were anesthetized via halothane inhalation, stunned and exsanguinated by severing the major blood vessels. [40] The trachea was removed and kept in ice-cold PSS until dissection. Tracheal rings of 2 mm width were cut with a single edge razor blade and the rings suspended on loops attached to the micrometer and force transducer system described above. The rings were stretched for at least two hours and pre-contracted with K+ and ACh before experimentation was begun, as described above. The rings were stimulated with 1 µM acetylcholine (Ach) and relaxed with 30 nM isoproternol (IP) in the presence or absence of 500 µM ascorbate. All tissues were weighed to the nearest 0.1 mg at the conclusion of the experiment.

### *In vivo* experiments

Tests of the enhancement effect of Asc on beta-adrenergic relaxation of pulmonary tissue were tested on horses and then sheep. The effect of Asc was tested in six horses with heaves, an asthma-like inflammatory obstructive airway disease similar to chronic obstructive pulmonary disease. The main cause of airway obstruction in heaves is cholinergically-mediated bronchospasm that is provoked by feeding hay, the dust from which initiates inflammation and airway obstruction [41]. In unsedated horses, an esophageal balloon connected to a pressure transducer and physiograph measured esophageal pressure which reflects pressure in the pleural cavity [42]. The severity of obstruction was evaluated from the change in pleural pressure during tidal breathing (ΔPpl_max_) and experiments were conducted on unsedated horses when ΔPpl_max_ was greater than 20 cm H_2_O [43]. The aerosol of ascorbate or vehicle generated by an ultrasonic nebulizer was delivered to the horse by means of a non-rebreathing valve and facemask. Albuterol was administered from a metered dose canister by means of a commercially available inhaler designed for horses (Torpex, Boehringer-Ingelheim Animal Health, St. Joseph, MO) [44], [45]. In a randomized crossover experiment, 6 heaves-affected horses were treated with vehicle or ascorbate (1 ml/min of 15 mg/ml Asc solution by nebulizer for 4 minutes) before administration of albuterol (120, 240, 360, and 720 micrograms). The ΔPpl_max_ was measured at baseline, 15 minutes after the end of vehicle/ascorbate administration and 15 minutes after each dose of albuterol and the data were expressed as the Ppl_ratio_, i.e. the ratio of ΔPplmax after drug dose to ΔPpl_max_ at baseline. At the conclusion of the experiments, the horses were given atropine (0.02 mg/kg IV) to test for reversibility of airway obstruction.

A total of 6 sheep (31–43 kg), with documented airway hypersensitivity to *Ascaris suum* antigen were used. The sheep were conscious and were restrained in a modified shopping cart in the prone position with their heads immobilized. Anesthesia of the nasal passages with topical 2% lidocaine was achieved and a balloon catheter was advanced through one nostril into the lower esophagus. The animals were intubated with a cuffed endotracheal tube through the other nostril as previously described. [46]–[48] Breath by breath determination of mean pulmonary airflow resistance (RL) was measured with the esophageal balloon technique described extensively. [6]–[8] The mean of at least 5 breaths, free of swallowing artifact, were used to obtain RL in cmH_2_O×L ^−1^×sec. All aerosols were generated using a disposable medical nebulizer (Raindrop^R^, Nelcor-Puritan Bennett, Carlsbad, CA). Aerosols were delivered at a tidal volume of 500 ml and a rate of 20 breaths per minute [46]–[48]. RL was measured at baseline and then immediately after 10 breaths of 2.0% w/v carbachol. The sheep then received a specified number of breaths of aerosol albuterol or ascorbate + albuterol at 15 min, 30 min, 1 h and 2 h after the carbachol challenge. RL was measured immediately after each treatment. A control trial was conducted in the same manner except that following carbachol challenge ascorbate alone (10 breaths of 15 mg/ml Asc solution) was given at each time point.

### Isolated receptor experiments

The availability of human beta 2 adrenergic receptor (Sigma) allowed direct experiments of the effect of Asc on the receptor. The human adrenergic receptor (AR) is available at 31.4 µM in a solution of 50 mM Tris, 10% glycerol and 1% BSA at pH 7.4. Ascorbate was prepared as a 500 µM stock in 20 mM sodium phosphate buffer at pH 7.4. Triplicate samples of 250 µl containing 0, 10, 30 and 100 µM Asc ± 1.26 µM receptor were prepared. The amount of adrenergic receptor buffer and phosphate buffer were constant in all samples. 200 µl of each sample were added to a 96 well plate and spectra collected from 190–310 nm in 1 nm increments during a series of repeated readings over a period of 2 hours in a Spectramax spectrophotometer. Difference spectra were used to measure the effect of the receptor on ascorbate over time. The data obtained here were compared with similar data obtained in a previous set of experiments reported in Dillon, et al. [4] using Angiotensin II transfected membrane (A2M) and untransfected membrane (UTM), which were obtained from Novascreen Biosciences (Hanover, Maryland) and prepared identically to the AR.

### Ascorbate oxidation

Since adrenergic receptors alter the rate of Asc oxidation in the spectrophotometer, we sought to ascertain the basal Asc oxidation rate in the spectrophotometer compared with the ambient Asc oxidation rate on the counter top. Asc oxidation can be measured in the spectrophotometer, using the ascorbate peak centered at 266 nm. As Asc is oxidized to DHA, this peak decreases, since DHA does not absorb at this wavelength. Ascorbate oxidation rates were measured using a Spectramax scanning spectrophotometer. 200 µl samples of ascorbate (0.1 mM stock in 20 mM sodium phosphate) were added to each of three wells in a crystal plate and scanned in the spectrophotometer. Every three minutes, another set of three wells in the plate were filled with 200 µl of ascorbate and the plate (including the previous sets of three wells) scanned again. Altogether, 24 wells were scanned from 230–300 nm at 2 nm increments. The Asc oxidation rate was determined by measuring changes in the Asc peak at 266 nm. The rate at which Asc oxidized during exposure to UV light in the spectrophotometer was compared with the rate at which Asc oxidized under ambient conditions. The ambient Asc oxidation rate was calculated using the initial absorbance of the fresh addition of Asc to the different wells, since prior to the initial measurement all their oxidation had taken place outside the spectrophotometer. The ambient temperature was 23.9C and the temperature inside the spectrophotometer was 24.8C. Rate constants were calculated from a linearized least-squares fit to an exponential decay using the Axum data processing software. In each case, a correlation coefficient for the rate constant was determined.

### Peptide Studies

A series of overlapping peptides (Table 1) synthesized by the Macromolecular Synthesis Facility at Michigan State University were tested for Asc binding using the same UV spectroscopy methods described above. The peptides synthesized included three spanning the first extracellular loop of the beta 2 adrenergic receptor (amino acids 89–99, 97–106, and 103–113) and three from the insulin receptor as controls. The best fit of the data to the Hill equation was used to determine the apparent dissociation constant K_da_. The actual K_d_s in Table 1 are calculated from K_d_ = K_da_−0.5[peptide concentration]. The effect of the peptides on Asc oxidation was measured using 50 µM peptide and 62.5 µM Asc in 50 mM potassium phosphate at pH 7.4. The Asc absorbance was measured at 266 nm over time in the presence and absence of the 89–99 and 97–106 peptides.

### Statistics

For repeated measures in which only two tests, with and without ascorbate, were performed on the same tissue or animal, a two-way ANOVA without replication was performed [49]. For the tests of relaxation and fade in porcine tracheal strips (Figures 1 and 2), each ascorbate/epinephrine concentration effect was individually compared with the relaxation and fade caused by epinephrine alone. Similarly, the reversal of isoproterenol fade (Figure 6) compares subsequent forces to the force immediately preceding the IP application. The ANOVA for these comparisons is mathematically equivalent to a t-test. The two-way ANOVA without replication was used to determine differences in the dose-response curves in the presence and absence of ascorbate for the effects of epinephrine on porcine trachealis force/cross-sectional area (Figure 3), isoproterenol-induced tachyphylaxis of guinea pig tracheal rings (Figure 5), and albuterol relaxation of sheep (Figures 7 and 8) and horse (Figure 9) airways. For comparison of ascorbate oxidation in the presence of peptides, a two-way ANOVA with replications (N = 3 at each time), was performed. For all tests, p<0.05 value was considered indicative of a difference between the tested groups.
